# SUMO-1 possesses DNA binding activity

**DOI:** 10.1186/1756-0500-3-146

**Published:** 2010-05-26

**Authors:** Sebastian Eilebrecht, Caroline Smet-Nocca, Jean-Michel Wieruszeski, Arndt Benecke

**Affiliations:** 1 Institut des Hautes Études Scientifiques, 35 route de Chartres, 91440 Bures-sur-Yvette, France; 2 Institut de Recherche Interdisciplinaire - CNRS USR3078 - Université Lille I - Université Lille II, 50 Av de Halley, 59658 Villeneuve d'Ascq, France; 3 Unité de Glycobiologie Structurale et Fonctionnelle, CNRS UMR8576 - Université de Lille1, Villeneuve d'Ascq, France

## Abstract

**Background:**

Conjugation of small ubiquitin-related modifiers (SUMOs) is a frequent post-translational modification of proteins. SUMOs can also temporally associate with protein-targets via SUMO binding motifs (SBMs). Protein sumoylation has been identified as an important regulatory mechanism especially in the regulation of transcription and the maintenance of genome stability. The precise molecular mechanisms by which SUMO conjugation and association act are, however, not understood.

**Findings:**

Using NMR spectroscopy and protein-DNA cross-linking experiments, we demonstrate here that SUMO-1 can specifically interact with dsDNA in a sequence-independent fashion. We also show that SUMO-1 binding to DNA can compete with other protein-DNA interactions at the example of the regulatory domain of Thymine-DNA Glycosylase and, based on these competition studies, estimate the DNA binding constant of SUMO1 in the range 1 mM.

**Conclusion:**

This finding provides an important insight into how SUMO-1 might exert its activity. SUMO-1 might play a general role in destabilizing DNA bound protein complexes thereby operating in a bottle-opener way of fashion, explaining its pivotal role in regulating the activity of many central transcription and DNA repair complexes.

## Background

Ubiquitin and small ubiquitin-like modifiers (SUMOs) form a family of structurally related proteins that often become covalently attached to other cellular factors. This post-translational modification of proteins, usually on lysine residues, with ubiquitin and SUMOs changes the functional properties of their targets [1-15]. Both, the regulation of the target's enzymatic activity and the modification of interaction patterns with third partners such as proteins and DNA have been described. Thereby, sumoylation apparently also provides a means to recruit SUMO interacting proteins via their SUMO binding motifs (SBMs) to the sumoylated target [16]. Furthermore, sumoylation and SUMO association have been found to influence other post-translational modifications such as phosphorylation. Sumoylation is an energy-dependent process carried out by a dedicated enzymatic machinery which is similar in composition and activity to the ubiquitination apparatus [2-7,9,10]. SUMO-specific proteases (SENPs) can reverse the process of sumoylation. Sumoylation has an essential role in most organisms. Deletion or inactivation of SUMOs or enzymes involved in the sumoylation process lead to severe growth defects in yeast or embryonic lethality in mice [2,3,8]. It is likely that lethality is the effect of deregulation of many different activities rather than only a single essential function given the variety of processes that involve sumoylation and SUMO binding [1-15].

Amongst the most prominent pathways which have been identified to be regulated by sumoylation and SUMO non-covalent interactions are transcriptional regulation and genome maintenance pathways [2,12-15,17]. Known targets for sumoylation include the transcription factors p53, HSF1, and STAT1. Transcription associated proteins such as the histone deacetylases (HDACs), or the co-repressors Daxx and CtBP are also found to be modified by SUMOs [14,15]. Particularly interesting is the role of sumolyation in the modification of the promyelocytic leukemia protein (PML) and the oncogenic chromosomal t15:17 translocation product PML-RARα, as SUMO modification and association has been shown to exert major effects on nuclear protein localization, stability, and activity. Similar effects have been observed on other transcription factors [18,19]. As a general theme, sumoylation of transcription associated proteins seems to have a negative effect on the promoter activity of target genes [20-23]. SUMO, by providing additional protein-protein interaction surfaces, predominantly recruits negative regulators of transcription, or sequesters transcription factors by an unknown mechanism into sub-nuclear domains devoid of transcription activity [2-15,20-23].

Another group of cellular pathways heavily influenced by sumoylation and SUMO binding are the genome maintenance systems [17]. Genetic manipulations in yeast have allowed to uncover a wealth of genome maintenance defects upon inactivation of different components of the SUMO system essentially involving all aspects of DNA damage response, replication, and chromosomal stability [2-15]. The importance of regulation of genome maintenance pathways by sumoylation has been confirmed for higher eukaryotes, albeit neither the targets nor the effects and still less the molecular mechanisms involved are known [2-15].

In this context the recent finding of sumoylation and SUMO association of Thymine-DNA Glycosylase (TDG) and the regulatory effects that SUMO proteins exert on this enzyme are of particular interest [24-26]. TDG is part of the base-excision DNA repair (BER) machinery targeting G:U and G:T mispairs in DNA. Indeed, these mismatches frequently occur on double-stranded DNA after spontaneous or catalytically-mediated hydrolysis of cytosine or C^5^-methylated cytosine leading to uracil and thymine, respectively. TDG and TDG-like activities are crucial for both, the avoidance of point-mutations and the maintenance of methylation patterns. SUMO conjugation and association have been well studied in the case of TDG, and lead to the description of partial structures of TDG-SUMO complexes, and important regulatory roles of SUMO proteins in the specification of TDG repair activity [24-26]. The fine structure analysis of the regulatory domain of TDG [27], has thereby allowed to better investigate the effect of SUMO-1 binding to and SUMO-1 covalent modification of TDG [28]. During the course of these works we made the observation that SUMO-1 can specifically interact with DNA and compete with other DNA binding proteins reported here.

## Results and Discussion

Based on our previous fine-structure analysis of the TDG regulatory domain (RD) dynamics of TDG [27], we started studying also different TDG SUMO complexes in the presence or absence of DNA using nuclear magnetic resonance. We thereby have uncovered a competition mechanism by which SUMO prevents or destabilizes RD DNA interactions. Such a competition can either be explained by a steric hindrance mechanism, or by direct competition for DNA binding [28].

**Figure 1:**
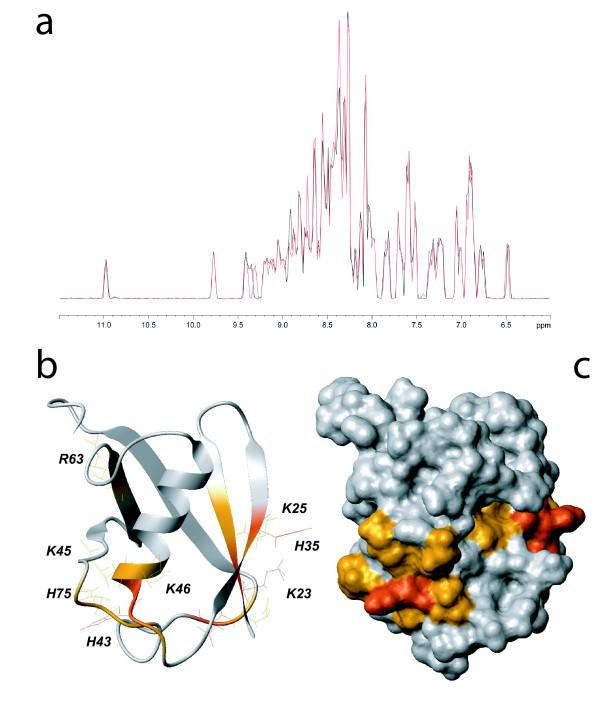
**SUMO-1 DNA interaction detected using NMR**. (A) Comparison of 1H projections of the 15N-1H HSQC spectra of 15N-SUMO-1 (black) and 15N-SUMO-1 at 20 μM with 135 μM of 25-mer double-stranded DNA containing a G:U mismatch (red). (B) SUMO-1 (PDB ID 2ASQ) ribbon representation in grey. SUMO-1 residues implicated in DNA binding are colored as a function of their chemical shift variations: red for Δδ > 0.06 ppm and orange for 0.02 < Δδ < 0.06 ppm. (C) SUMO-1 solvent accessible surfaces colored as described for B.

We thus investigated the ability of SUMO-1 to directly interact with DNA. By acquiring a HSQC spectrum on 15N-labeled SUMO-1 at 20 μM in presence of 135 μM of the same DNA substrate containing a central G:U mismatch which had been used in the experiments leading to the hypothesis of SUMO-1 DNA binding (5'-GCTTCGAGTACCUGTGGAACCTATC-3') we observe chemical shift perturbations for several SUMO-1 resonances (Figure 1A) concerning mainly positively charged residues and their neighborhood. They encompass the K23 and K25 residues of the β1 strand, H35 of the β2 strand, H43 of the β2/α1 loop, K45, K46 and K48 of the α1 helix, R63 of the β4 strand and finally H75 from the β4/β5 loop (Figure 1B, C). Importantly, other positively charged and solvent-accessible residues such as K37, K39 in the immediate vicinity, as well as K10, K19, and K20 in the N-terminus of SUMO-1 are not perturbed, indicating specificity of the observed DNA interaction with before mentioned sites. Furthermore, the residues for which DNA interactions are observed form a continuous patch along the SUMO-1 surface.

**Figure 2:**
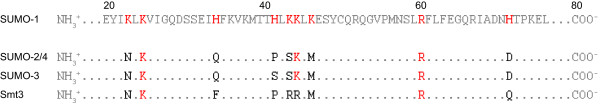
**Conservation of SUMO-1 residues implicated in DNA binding amongst SUMO family members**. Residues 20-80 of human SUMO-1 are shown, the residues colored in red display chemical shift variations Δδ > 0.02 ppm when in contact with DNA. For the other SUMO family members only the residues at the corresponding positions are shown.

Interestingly, only K25 and R63 are conserved amongst all SUMO family members, indicating that DNA binding activity might be restricted to SUMO-1 (Figure 2).

**Figure 3:**
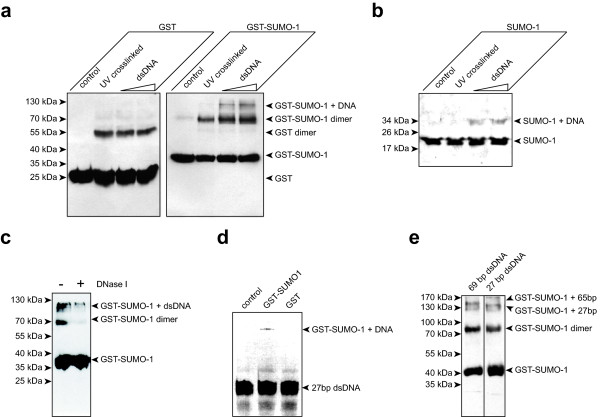
**UV cross-linking experiments confirm SUMO-1 DNA interaction**. (A) 100 ng of GST or GST-SUMO-1 were exposed in the absence or presence of 200 ng or 500 ng of dsDNA (69-mer) to UV (312 nm, 200 W) for 10 min. After 12.5% SDS-PAGE and western-blotting protein complexes were detected using a GST antibody and compared to non-exposed samples ('control'). (B) 100 ng of SUMO-1 were exposed in the absence or presence of 400 ng or 1 μg of dsDNA (27-mer) to UV (312 nm, 200 W) for 10 min. After 12.5% SDS-PAGE and western-blotting protein complexes were detected using a SUMO-1 antibody and compared to non-exposed samples ('control'). (C) The GST-SUMO-1 + DNA complex is sensitive to DNase treatment. Experiments as in A using 200 ng of dsDNA were post cross-linking and prior SDS-PAGE incubated with or without DNaseI (2 h, 37°C). (D) The high molecular weight bands formed by GST-SUMO-1 in the presence of DNA can also be shown to contain DNA using a fluorescent dsDNA. (E) Sequence independence of SUMO-1 DNA interaction is demonstrated using two different dsDNAs and detected after 10% SDS-PAGE.

**Figure 4:**
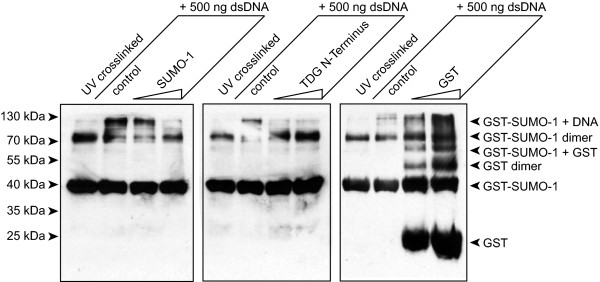
**Competition of SUMO-1 DNA interaction by TDG N-terminus**. GST-SUMO-1 DNA complexes were incubated with increasing amounts of free SUMO-1, the TDG N-terminus or GST for the UV-crosslinking experiments carried out under the conditions described for Figure 3. The double stranded oligonucleotide used was the 65-mer. "Control" refers to the GST-SUMO-1 alone. Roughly 2 and 5 molar excess of competitor proteins were used.

SUMO-1 DNA complexes can also be detected using UV-crosslinking experiments. Figure 3A illustrates the formation of GST-SUMO-1 DNA complexes specific to the presence of a 65 bp double-stranded mismatch-free DNA oligonucleotide (5'-CGGATCCCAACTCCAGGAAGGAAACCA AGCGATTGGTTTCCTTCCTGGAGTTGTTTTTCCCGGGT-3'). Free GST protein does not interact with DNA. Note that we used GST fusion proteins, despite their capacity to dimerize which slighly complicates the analysis of the results (ref, Figure 3, 4), in order to provide for a negative control and concomitant detection with a single antibody in order to exclude biases. Indeed, also wild-type SUMO-1 protein lacking a GST-tag can be shown to bind the 27 bp double-stranded DNA oligonucleotide (5'-CGGGGCCGGGGCGGGCCGCGCAAGCAG-3') (Figure 3B) in similar experiments. As confirmed using DNaseI digestion (Figure 3C) the high molecular weight band attributed to GST-SUMO-1 DNA complexes contains DNA. DNaseI treatment destabilizes GST-SUMO-1 dimer formation, which conincides with a slight increase of GST-SUMO-1 dimer formation upon addition of increasing amounts of double-stranded DNA (Figure 3A). These observations can be explained by dsDNA stabilizing the GST-SUMO-1 dimers created through homo-association via their GST tags. A dimerization of non-tagged SUMO-1 protein is not observed (Figure 3B). Our findings are further substantiated when an unrelated, double-stranded, 3'-NH-AlexaFluor488^® ^labeled oligonucleotide (5'-AGCTTCGAGTACCCGTGGAACCTATCG-3'), is used in the UV-crosslinking experiments and the reaction products are analyzed for their fluorescence (Figure 3D). Furthermore, as the sequences of the 65-mer and the 27-mer are unrelated and also differ from the 25-mer G:U mismatch oligonucleotide used in the NMR experiments, these experiments establish sequence independence of the SUMO-1 DNA interaction. We then compared the relative retention of the GST-SUMO-1 complexes formed with either the 65-mer or the 27-mer DNA strands. The complex formed in the presence of the shorter 27 mer dsDNA indeed migrates faster than the one corresponding to the 65-mer (Figure 3E).

Finally, as we propose elsewhere a direct competition between SUMO-1 and the TDG regulatory domain for DNA binding [28], we thought to directly demonstrate such competition. To this end we incubated the GST-SUMO-1 DNA mixture with increasing amounts of either untagged SUMO-1 or the human TDG N-terminus (aa1-111), and as a control free GST. As shown in Figure 4, both free SUMO-1 and TDG N-terminus compete with GST-SUMO-1 for DNA binding when present in two- or five-fold molar excess in the reaction. Free GST does not compete with DNA complex formation. These experiments do not only confirm the specificity of the SUMO-1 DNA interaction but also lend further support to the previously reported capacity of the TDG RD to bind to DNA, and the possible competition between both entities for DNA during TDG mediated base-excision repair.

A complex pattern of chemical shift variations was previously observed upon NMR-based titration of 15N-labeled TDG-N with DNA exhibiting different dissociation constants related to various sites of the TDG-N protein [27,28]. Relatively low dissociation constants (about 20 μM) have been measured for the TDG-N/DNA complex around K64, K83/K84/K87 and K103/K105 sites. For the intertwining regions, a higher Kd of 150 μM was estimated, and SUMO-1 competes more efficiently with TDG-N in these latter regions for DNA binding. Based on the ability of SUMO-1 to compete with TDG-N to bind DNA, as observed here with both NMR and cross-linking experiments, the dissociation constant of the SUMO-1/DNA complex was estimated in the range of 1 mM.

## Conclusion

We have this established here using NMR and UV-crosslinking experiments that SUMO-1 possesses specific but sequence-independent DNA binding activity. The amino-acids involved in this interaction have been identified, and are only weakly conserved in identity or charge amongst the different members of the SUMO family, indicating an important potential difference between different SUMO members.

More importantly, the fact that SUMO-1 can bind DNA, and the observation that this DNA binding is in competition with the regulatory domain of TDG, allows to explain how SUMO-1 exerts its activity on TDG [28]. The proposed salvage mechanism, allowing the removal of TDG from DNA after the glycosylase reaction, might also be a general scheme of SUMO-1 action on other DNA binding activities such as transcription factors and co-factors or the genome maintenance proteins known to be sumoylated or interact with SUMO-1 through SBMs [2-23]. SUMO-1 might play a general role in destabilizing DNA bound protein complexes thereby operating in a bottle-opener way of fashion [18,19].

## Materials and methods

### Expression and purification of recombinant SUMO-1 and TDG N-terminus

SUMO-1 and TDG-N proteins were overexpressed in BL21(DE3) strain as GST fusion protein. Bacteria were grown at 37°C in M9 minimal medium reconstituted with 2 g/l glucose, 1 g/l ^15^N-labeled ammonium chloride, 1 mM MgSO_4_, MEM vitamin cocktail (Sigma) (or in LB medium for the production of unlabeled proteins) and 100 mg/l ampicilline. Protein expression was induced overnight at 20°C following 0.5 mM IPTG addition. Cells were harvested and resuspended in extraction buffer (PBS, 10% glycerol, 1% Triton X-100, 10 mM EDTA, 2 mM DTT) complemented with a protease inhibitor cocktail (Complete, Roche). Cell lysates were obtained by incubation of 0.25 mg/ml lysozyme with the cell suspension in extraction buffer complemented with RNase and DNase followed by brief sonication steps. The soluble extract was isolated by centrifugation. SUMO-1 GST-fusion protein and wild-type GST were purified on a Glutathione Sepharose resin (GE Healthcare). Soluble extracts were incubated for 3 hours at 4°C with 25 to 100 μl resin per milliliter of soluble extracts. Unbound proteins were extensively washed away with a GST wash buffer (PBS, 5% glycerol, 1% Triton X-100, 10 mM EDTA), and TDG-N protein was eluted by digestion with Precission Protease using 25 μg/ml of resin (GE Healthcare) in one bead volume of elution buffer (50 mM Tris-Cl pH 8.0, 150 mM NaCl, 2% glycerol, 0.1% NP-40, 10 mM EDTA, 5 mM DTT). The reaction was allowed to proceed at 4°C for 20 hours. Then beads were eluted twice with one bead volume of elution buffer. GST-SUMO-1 was eluted in one bead volume of elution buffer (50 mM Tris-Cl pH 8.0, 200 mM NaCl, 5 mM EDTA, 5 mM DTT) containing 10 mM of reduced (L)-glutathione and SUMO-1 was obtained by an overnight incubation with 1 unit of thrombin per mg of protein at room temperature. Proteins were concentrated and purified by gel filtration on a preparative Superdex75 column (GE Healthcare) equilibrated in NMR sample buffer. Proteins were concentrated to obtain final concentrations of 500 μM for SUMO-1. The protein homogeneities were verified on denaturing polyacrylamide gel, the molecular mass and isotopic labeling by MALDI-TOF mass spectrometry.

### NMR analysis of the interaction of SUMO-1 with dsDNA

For the DNA substrates, the sequences of the 5'-3' strands are GAATTCGATAGGTTCC ACGGGTACTCGAAGCGGATCC and GATAGGTTCCACGGGTACTCGAAGC for the 37- and the 25-mer, respectively. Annealing of oligonucleotides was performed by heating 1 mM solutions for 5 min at 100°C and cooling down the mixtures slowly to room temperature to obtain double-stranded 25-mers. These solutions were lyophilized and dissolved at 50 μM final concentration. Interactions of SUMO-1 with DNA was performed with the 25-mer double-stranded DNA substrate with 20 μM of ^15^N-labeled SUMO-1 and 135 μM DNA in a buffer constituted by 100 mM NaiPO_4 _pH 6.6, 1 mM DTT and 1 mM EDTA. ^15^N-^1^H HSQC spectra were acquired for each condition with ^1^H and ^15^N spectral windows of 16 and 36.5 ppm, respectively, and with 256 scans. The chemical shift perturbations of individual resonances were calculated using the following Eq 1.(1)

### DNA oligonucleotides, hybridization, DNase I digest

The sense sequence of the double-stranded oligonucleotides used was 5'-CGGAT CCCAA CTCCA GGAAG GAAAC CAAGC GATTG GTTTC CTTCC TGGAG TTGTT TTTCC CGGGT-3' (65-mer) and 5'-CGGGG CCGGG GCGGG CCGCG CAAGC AG-3' (27-mer). Each sense oligonucleotide was heated to 95°C for 10 minutes with equal amounts of the corresponding antisense oligonucleotide and cooled down to 20°C within one hour for appropriate hybridization.

DNase digest was performed using 1 Unit of DNase I (Fermentas) incubating for 2 hours at 37°C prior to UV crosslink.

### UV crosslink, Competition

Approximately 100 ng of GST fusion protein was incubated with increasing amounts (200 ng, 500 ng) of double-stranded DNA for 10 minutes at 37°C in NMR buffer (100 mM Na2PO4 pH 6.6, 1 mM EDTA, 1 mM DTT). GST fusion protein alone was used as a control. The sample was cooled down immediately for 5 minutes on ice and exposed to UV light for 10 minutes (TFX-20.M, 15 W, 312 nm). To check for effects of the UV crosslink to the protein alone, non-crosslinked GST fusion protein was used as a control.

To investigate DNA binding activity of SUMO-1 wild type protein, approximately 100 ng of SUMO-1 was incubated with increasing amounts (400 ng, 1000 ng) of double-stranded DNA and treated as above-mentioned.

To analyze competition of DNA-binding, 100 ng of GST-SUMO1 was incubated with 500 ng of double-stranded DNA in presence of increasing amounts of wild type SUMO1 or TDG-N-Terminus (200 ng, 400 ng) prior to UV crosslink.

### Western blot

Western blot analyses were performed as described previously [29] using mouse anti-GST antibody (Invitrogen) and HRP-coupled goat anti-mouse antibody (Invitrogen) as recommended by the manufacturer. For detection of SUMO-1 a rabbit anti-SUMO-1 antibodiy(Pierce) and a HRP-coupled goat anti-rabbit antibody (Cell Signaling) was used as recommended by the manufacturer.

## Competing interests

The authors declare that they have no competing interests.

## Authors' contributions

CSM: Protein purification, NMR; SE: Protein purification, EMSA, cross-linking; JMW: Technical assistance NMR; AB: Supervision, writing of manuscript. All authors have read and approved the final manuscript.
